# Source memory errors associated with reports of posttraumatic flashbacks: A proof of concept study

**DOI:** 10.1016/j.cognition.2012.05.002

**Published:** 2012-08

**Authors:** Chris R. Brewin, Zoe Huntley, Matthew G. Whalley

**Affiliations:** Clinical, Educational & Health Psychology, University College London, UK

**Keywords:** PTSD, False memory, Flashbacks, Trauma, Imagery

## Abstract

Flashbacks are involuntary, emotion-laden images experienced by individuals with posttraumatic stress disorder (PTSD). The qualities of flashbacks could under certain circumstances lead to source memory errors. Participants with PTSD wrote a trauma narrative and reported the experience of flashbacks. They were later presented with stimuli from flashback and non-flashback parts of their narrative, mixed with foils from the narrative of another participant, and judged whether they belonged to their own narrative. They also reported whether stimuli elicited a flashback during this recognition test. Overall reporting a flashback at test was associated with significantly better recognition performance. Flashbacks were occasionally reported to foil stimuli, which were then likely to be wrongly attributed to the person’s own narrative. This provides proof of concept of a cognitive mechanism that could potentially account for some cases of false trauma memories.

## 1 Introduction

It is generally accepted that recovered memories of trauma may or may not correspond to actual events (Geraerts et al., 2009). Therapeutic suggestion may account for some instances of false recall, but memory recovery often occurs outside therapy (Brewin, 2012), requiring alternative explanations. ‘Flashbacks’ are a symptom of posttraumatic stress disorder (PTSD) and are often observed in patients recovering memories of traumatic events (Andrews et al., 2000; Schooler, Bendiksen, & Ambadar, 1997). They consist of a type of intense involuntary memory involving repeated reliving of the traumatic event, accompanied by marked sensory detail and emotional arousal (Brewin, 2007, 2011). High levels of sensory detail are normally associated with true rather than false recollection (Schooler, Gerhard, & Loftus, 1986; Suengas & Johnson, 1988), suggesting that flashbacks are likely to be associated with previously experienced items and events. However, if apparent recollections are in fact false, the occurrence of a flashback might lead them to be incorrectly labelled as true. This study attempted to establish proof of concept of this hypothetical cognitive mechanism for producing false recall.

In previous studies flashbacks have been characterised by traumatic events being reexperienced in the present rather than the past (Brewin, Gregory, Lipton, & Burgess, 2010; Ehlers, Hackmann, & Michael, 2004). They can vary from relatively mild (there is a transient sense of the event reoccurring in the present) to extreme (the person loses all connection with their current autobiographical self and present surroundings while reexperiencing the memory). They are not typically reported in healthy participants exposed to trauma (Brewin, 2011).

True memories are thought to possess on average more detail than false memories, particularly sensory details involving sights, sounds, and smells (Marche, Brainerd, & Reyna, 2010; Schooler et al., 1986; Suengas & Johnson, 1988; Vrij, 2005), and are more likely to be associated with a sense of recollection (Conway, Collins, Gathercole, & Anderson, 1996; Heaps & Nash, 2001) and emotional intensity (Heaps & Nash, 2001; Laney & Loftus, 2008). Although many of these studies could be criticised for confounding the truth or falsity of memories with memory strength, the literature suggests that stimuli that elicit reports of a flashback at recall are, if true memories, more likely to be correctly labelled as such. More rarely, however, such apparent recollections may be false, in which case the occurrence of a flashback may lead to them being incorrectly labelled as true. The existence of this hypothetical effect has never, to our knowledge, been demonstrated.

Individuals suffering from PTSD first wrote a detailed narrative account of their main traumatic event, and identified flashback and non-flashback sections. Single words and phrases were then extracted from the two sections of each person’s narrative and presented to them one week later. Following previous work (Barclay & Wellman, 1986), these were intermixed with foils supplied by a second individual with PTSD . The task, which combined item and source memory, was to recognise whether or not each word or phrase belonged to their own narrative. Participants reported at the end of the testing session whether each word or phrase had elicited a flashback or not. Words and phrases that elicited a flashback, either during narrative production or at recall, were expected to be rated at recall as more arousing and of greater negative valence, and to be better recognised. We also investigated whether there were any instances in which participants reported flashbacks to stimuli from another person’s narrative, and predicted that they should be more likely to incorrectly classify any such words or phrases as their own.

## 2 Method

### 2.1 Participants

There were 10 participants (3 men), all meeting diagnostic criteria for current PTSD when assessed with the Structured Clinical Interview for DSM-IV (First, Williams, & Spitzer, 1997). They had experienced a range of traumas, including involvement in disasters or terrorist attacks (*n *= 3), interpersonal violence and robbery (*n *= 4), motor vehicle accident (*n *= 1), abduction by security services (*n *= 1), and witnessing of mother’s dying moments (*n *= 1). Their average age was 40.3 years (*SD* 9.6, range 28–57 years). Three were currently taking antidepressant medication. All participants gave written informed consent.

### 2.2 Measures

The Posttraumatic Diagnostic Scale (PDS) (Foa, Cashman, Jaycox, & Perry, 1997) is a widely-used self-report measure. Items measuring each of the 17 PTSD symptoms are rated for the past month on a 0–3 scale. In this sample the mean PDS score was 25.30 (SD = 5.85).

The Beck Depression Inventory 2 (Beck, Steer, & Brown, 1996) is a widely-used 21-item self-report measure of depression severity. It contains 21 items that are scored on a 4-point scale (possible range 0–63). Participants were instructed to rate their mood over the past week. In this sample the mean BDI score was 26.60 (SD = 10.66), indicating moderate depression (Kendall, Hollon, Beck, Hammen, & Ingram, 1987).

### 2.3 Procedure

Participants preparing to take part in an fMRI study (Whalley, Kroes, Rugg, & Brewin, submitted for publication) wrote an account of their traumatic event, starting from just before they knew something was wrong until the point where the event had resolved. Following Hellawell and Brewin (2002), flashbacks were defined for participants who then highlighted sections during the writing of which they had experienced flashbacks. Words identified by them as belonging to the flashback sections of their narrative were tabulated separately from non-flashback words and were rated for frequency (Kucera & Francis, 1967) and number of letters. Lists of 36 flashback words and 36 non-flashback words were created and matched to ‘master lists’ (see below) on word frequency and length. Phrases (typically 2–8 words long) were tabulated separately using the same procedure, and matched for length. Lists of 30 flashback and non-flashback phrases were matched to ‘master lists’ on number of letters and words per sentence.

Two ‘master lists’ were generated from other participants with PTSD to provide comparable stimuli unrelated to the participant’s own traumatic event. One was from an individual who had survived the July 7th 2005 London bombings, and one from a survivor of the December 2004 Asian Tsunami. Certain words such as ‘blood’ or ‘helpless’ were common to many narratives, but words on the master list were substituted on a case-by-case basis whenever overlap was identified.

During a second testing session that occurred approximately one week later stimuli from participants’ own list and one of the master lists were presented randomly on a screen at a distance of approximately 50 cm while participants were being scanned. Single words were presented in the centre of the screen in uppercase 40pt, Arial font, and phrases in lowercase 30pt Arial font. The task in each case was to identify whether each word came from their narrative or from the narrative of another participant and to respond as quickly and accurately as possible using a button-box. For both tasks (task 1: words; task 2: sentences) the presentation of an item was preceded by an asterisk (*) for 500 ms, followed by the item for (1000 ms in task 1, 1700 ms in task 2), followed by a fixation cross for 2000 ms. These sequences of events gave stimulus onset asynchronies for tasks 1 and 2 of 3500 ms, and 4200 ms respectively. At the end of this session participants were shown a list of the stimuli and were asked to identify which, if any, items had led to them having flashbacks during the task. Participants were then given lists of all the stimuli they had seen during the test and were asked to rate each item on separate 7-point Likert scales measuring valence (scale anchored with [1] Unpleasant and [7] Pleasant) and arousal (scale anchored with [1] Low arousal and [7] High arousal).

## 3 Results

### 3.1 Reliability and validity of flashback judgments

As shown in Table 1, experiencing a flashback during the recognition test was reported to approximately half the stimuli which had elicited a flashback during production of the participant’s own narrative (Own Flashback items) and to a quarter of stimuli that had not done so (Own Non-flashback items). Experiencing a flashback to items from the control PTSD narrative (Other Flashback and Other Non-flashback items) was rare. We tested the reliability of flashback judgements with a 2 (Content: Words vs. Phrases) × 4 (Narrative Section: Own Flashback, Own Non-flashback, Other Flashback, Other Non-flashback) ANOVA on the percentage of flashbacks endorsed during the recognition test. After Greenhouse-Geisser correction for departure from sphericity there were significant effects of Content (*F*(1, 9) = 6.54, *p* < .05, partial eta squared = .42), Narrative Section (*F*(3, 27) = 21.97, *p* < .001, partial eta squared = .71), and the Content x Narrative Section interaction (*F*(3, 27) = 6.54, *p* < .01, partial eta squared = .42).

A follow-up 1-way ANOVA showed that, collapsing across words and phrases, the numbers of flashbacks in all sections differed from each other significantly (Own Flashback 15.65, Own Non-flashback 9.00, Other Flashback 1.40, Other Non-flashback .50; *p* < .05). Follow-up *t*-tests showed that Own Flashback phrases led to a higher percentage of flashbacks than Own Flashback Words, *t*(9) = 3.13, *p* < .02, whereas Other Flashback words led to more flashbacks than Other Flashback phrases, *t*(9) = 3.69, *p* < .01. There were no differences between Own Non-flashback words and phrases, or between Other Non-flashback words and phrases, largest *t*(9) = 1.86, *p* > .05. As the data were not normally distributed the analyses were repeated using nonparametric Friedman and Wilcoxon tests for related samples, which yielded similar findings.

The validity of the flashback judgements was tested by examining ratings of valence and arousal. A 2 × 4 ANOVA on valence ratings showed a main effect of Narrative Section (after Greenhouse-Geisser correction *F*(3, 18) = 7.61, *p* < .02, partial eta squared .56) but no effect of Content (*F*(1, 6) = 2.36, *p* > .10), and no Content x Narrative Section interaction (*F*(3, 18) = 1.95, *p* > .10). Collapsing across Content, Own Flashback words and phrases (Mean = 1.94) were rated as more negative (*p* < .05) than all other sections (Own Non-flashback 3.25, Other Flashback 3.79, Other Non-flashback 4.00).

Ratings of arousal demonstrated an identical pattern, with a main effect of Narrative Section (after Greenhouse-Geisser correction *F*(3, 18) = 13.23, *p* < .01, partial eta squared .69) but no effect of Content (*F*(1, 6) < 1) and no Content x Narrative Section interaction (*F*(3, 18) = 1.29, *p* > .10). Collapsing across Content, Own Flashback words and phrases (Mean = 5.91) were rated as more arousing (*p* < .05) than all other sections (Own Non-flashback 4.56, Other Flashback 3.40, Other Non-flashback 3.08).

### 3.2 Association of flashbacks with recognition

A similar analysis was conducted on recognition accuracy scores (hit/correct rejection rate). Accuracy rates were too high to enable a separate test of misses. This showed no effect of Narrative Section (*F*(3, 27) = 1.16, *p* > .05), and no Narrative Section × Content interaction (*F*(3, 27) = 1.34, *p* > .05), but a main effect of Content (*F*(1, 9) = 80.64, *p* < .001, partial eta squared .90). Phrases (mean hit rate .89) were recognised significantly more accurately than single words (mean hit rate .75). A further ANOVA conducted on Own stimuli only tested whether Content, Narrative Section, and the occurrence of a flashback during test predicted accuracy. The results indicated that reporting a flashback during the recognition test (*F*(1, 8) = 13.52, *p* < .01, partial eta squared .63) was associated with better recognition performance. There were no main effects or 2-way interactions involving Content or having had a flashback during the original narrative, largest *F*(1, 8) = 2.64, *p* > .05. The 3-way interaction was significant (*F*(1, 8) = 11.90, *p* < .01, partial eta squared .60). Recognition was highest for phrases that had elicited a flashback both in the original narrative and at recall (mean hit rate .96), and was lowest for single words that had not elicited a flashback either in the original narrative or at recall (mean hit rate .69).

The main question concerned whether participants ever reported a flashback on presentation of a stimulus from the control PTSD narrative and, if so, what was the effect on recognition judgements. Seven participants reported one or more examples of this response, which occurred an average of 3.80 times (median 2, range 1–16, *SD* 5.14) during the course of the experiment. In this group the mean accuracy of these responses was compared with their mean accuracy when stimuli from the control narrative did not elicit a flashback. A paired t-test indicated that mean accuracy when a flashback was experienced (.29) was very low, with the stimuli being incorrectly judged as their own, compared to when a flashback was not experienced (.81). This difference was large in magnitude and highly significant, *t*(6) = 4.56, *p* = .004, *d* = 1.72. Removal of the single outlier who reported 16 examples of this response did not change the results.

## 4 Discussion

In this investigation flashback judgments showed moderate levels of reliability, clearly indicating the probabilistic nature of flashback elicitation by verbal cues. Consistent with previous research (Hellawell & Brewin, 2002; 2004), they also had valid external referents in the form of valence and arousal ratings, suggesting that participants with PTSD can meaningfully discriminate the occurrence of a flashback. Memory accuracy was not consistently better for stimuli that had elicited a flashback report during narrative production, a finding which may have been due to a lack of statistical power.

However, consistent with autobiographical memory research suggesting that true memories are characterised by greater emotion and greater sensory detail, reporting a flashback at test was associated with significantly greater recognition accuracy. At present it is not clear whether higher accuracy can be accounted for by greater arousal, greater negative valence, greater reliving, or some other characteristic.

Previous research on flashbulb memories has confirmed that recall for even highly emotional events can be inaccurate (Neisser & Harsch, 1992; Schmolck, Buffalo, & Squire, 2000). Emotional stimuli, relative to neutral stimuli, appear to produce a stronger sense of recollection even when they are not objectively remembered more accurately (Sharot, Delgado, & Phelps, 2004). In hypnosis research, the credibility of a suggested past-life experience was predicted by the subjective intensity of that experience (Spanos, Menary, Gabora, DuBreuil, & Dewhirst, 1991).

We extended these findings by demonstrating that on those rare occasions when foil items were associated with a powerful involuntary trauma image, the items were very likely to be misclassified. The effect size obtained was equivalent to the foil items being 8.5 times more likely to be classified as from the participants’ own narrative in the presence of a flashback than in its absence. These data provide the first proof of concept of a potential mechanism for explaining some occurrences of false recovered trauma memories in clinical samples. Although foil stimuli were selected for being thematically unrelated to the participant’s traumatic event, it is conceivable that they did in fact have some association with information in the underlying memory representation. Similarly, it has been argued that in the clinic false memories do not arise *de novo* but may be based in part on memories of real events (Mollon, 1998). Thus in many cases false memories may be better conceived of as substantially false (but partially correct) memories.

Among the limitations of the study is that the effect of presenting large numbers of personally-relevant cues in proximity to one another is unknown. Had presentation been interspersed with a greater amount of unrelated material, so that stimuli from the participant’s own narrative occurred more rarely, or had presentation not taken place inside a brain scanner, rates of reported flashback elicitation may have been very different. Replication is required using larger samples, different types of stimuli (including events rather than just words), and different types of eliciting context. The data do, however, add to our extremely limited knowledge concerning flashback reports and may provide a platform for better theorizing in the future. They also, more tentatively, add to existing research suggesting cognitive mechanisms that may sometimes mislead individuals recovering traumatic memories into wrongly classifying imaginary events as being part of their personal history (Brewin, 2012; Geraerts et al., 2009; Loftus & Davis, 2006).

## Figures and Tables

**Table 1:** Mean percentage of flashbacks, mean valence, mean arousal, and mean accuracy of recognition of single words and phrases from own and control narratives (standard deviations in parentheses).

Narrative Section	Percent flashbacks during recognition	Valence	Arousal	Recognition accuracy
*Single words*				
Own Flashback	41.42 (27.63)	2.13 (.88)	5.78 (.90)	.81 (.09)
Own Non-flashback	23.35 (20.89)	3.46 (.94)	4.25 (1.18)	.76 (.12)
Other Flashback	6.12 (6.26)	3.99 (1.17)	3.36 (1.32)	.71 (.13)
Other Non-flashback	1.11 (1.94)	3.95 (1.38)	3.16 (1.32)	.72 (.12)

*Phrases*				
Own Flashback	54.61 (32.78)	1.75 (.48)	6.05 (.51)	.89 (.09)
Own Non-flashback	31.97 (25.76)	3.04 (1.08)	4.87 (1.05)	.88 (.08)
Other Flashback	2.00 (4.49)	3.59 (1.62)	3.43 (1.47)	.88 (.08)
Other Non-flashback	2.00 (3.58)	4.06 (1.32)	2.99 (1.27)	.89 (.10)

N.B. Own flashback = own narrative items that elicited a flashback at production; Own Non-flashback = own narrative items that did not elicit a flashback at production; Other Flashback = control PTSD narrative items that elicited a flashback at production; Other Non-flashback = control PTSD narrative items that did not elicit a flashback at production.

## References

[b0005] Andrews B., Brewin C.R., Ochera J., Morton J., Bekerian D.A., Davies G.M. (2000). The timing, triggers and qualities of recovered memories in therapy. British Journal of Clinical Psychology.

[b0010] Barclay C.R., Wellman H.M. (1986). Accuracies and inaccuracies in autobiographical memories. Journal of Memory and Language.

[b0015] Beck A.T., Steer R.A., Brown G.K. (1996). Manual for the Beck Depression Inventory-II.

[b0020] Brewin C.R. (2007). Autobiographical memory for trauma: Update on four controversies. Memory.

[b0025] Brewin C.R. (2011). The nature and significance of memory disturbance in posttraumatic stress disorder. Annual Review of Clinical Psychology.

[b0030] Brewin C.R., Belli R.F. (2012). A theoretical framework for understanding recovered memory experiences. True and false recovered memories: Toward a reconciliation of the debate.

[b0035] Brewin C.R., Gregory J.D., Lipton M., Burgess N. (2010). Intrusive images in psychological disorders: Characteristics, neural mechanisms, and treatment implications. Psychological Review.

[b0040] Conway M.A., Collins A.F., Gathercole S.E., Anderson S.J. (1996). Recollections of true and false autobiographical memories. Journal of Experimental Psychology: General.

[b0045] Ehlers A., Hackmann A., Michael T. (2004). Intrusive re-experiencing in post-traumatic stress disorder: Phenomenology, theory, and therapy. Memory.

[b0050] First M.B., Williams J.B., Spitzer R.L. (1997). Structured Clinical Interview For DSM-IV Axis I Disorders (SCID-I).

[b0055] Foa E., Cashman L., Jaycox L., Perry K. (1997). The validation of a self-report measure of PTSD: The posttraumatic diagnostic scale. Psychological Assessment.

[b0060] Geraerts E., Lindsay D.S., Merckelbach H., Jelicic M., Raymaekers L., Arnold M.M. (2009). Cognitive mechanisms underlying recovered-memory experiences of childhood sexual abuse. Psychological Science.

[b0065] Heaps C.M., Nash M. (2001). Comparing recollective experience in true and false autobiographical memories. Journal of Experimental Psychology-Learning Memory and Cognition.

[b0070] Hellawell S.J., Brewin C.R. (2002). A comparison of flashbacks and ordinary autobiographical memories of trauma: Cognitive resources and behavioural observations. Behaviour Research and Therapy.

[b0075] Hellawell S.J., Brewin C.R. (2004). A comparison of flashbacks and ordinary autobiographical memories of trauma: Content and language. Behaviour Research and Therapy.

[b0080] Kendall P.C., Hollon S.D., Beck A.T., Hammen C.L., Ingram R.E. (1987). Issues and recommendations regarding use of the Beck Depression Inventory. Cognitive Therapy and Research.

[b0085] Kucera H., Francis W.N. (1967). Computational analysis of present-day American English.

[b0090] Laney C., Loftus E.F. (2008). Emotional content of true and false memories. Memory.

[b0095] Loftus E.F., Davis D. (2006). Recovered memories. Annual Review of Clinical Psychology.

[b0100] Marche T.A., Brainerd C.J., Reyna V.F. (2010). Distinguishing true from false memories in forensic contexts: Can phenomenology tell us what is real?. Applied Cognitive Psychology.

[b0105] Mollon P. (1998). Remembering trauma: A psychotherapist’s guide to memory and illusion.

[b0110] Neisser U., Harsch N., Winograd E., Neisser U. (1992). Phantom flashbulbs: False recollections of hearing the news about challenger. Affect and accuracy in recall: Studies of “flashbulb memories”.

[b0115] Schmolck H., Buffalo E.A., Squire L.R. (2000). Memory distortions develop over time: Recollections of the O.J. Simpson trial verdict after 15 and 32 months. Psychological Science.

[b0120] Schooler J.W., Bendiksen M., Ambadar Z., Conway M.A. (1997). Taking the middle line: Can we accommodate both fabricated and recovered memories of sexual abuse?. Recovered memories and false memories.

[b0125] Schooler J.W., Gerhard D., Loftus E.F. (1986). Qualities of the unreal. Journal of Experimental Psychology: Learning Memory and Cognition.

[b0130] Sharot T., Delgado M.R., Phelps E.A. (2004). How emotion enhances the feeling of remembering. Nature Neuroscience.

[b0135] Spanos N.P., Menary E., Gabora N.J., DuBreuil S.C., Dewhirst B. (1991). Secondary identity enactments during hypnotic past-life regression: A sociocognitive perspective. Journal of Personality and Social Psychology.

[b0140] Suengas A.G., Johnson M.K. (1988). Qualitative effects of rehearsal on memories for perceived and imagined complex events. Journal of Experimental Psychology-General.

[b0145] Vrij A. (2005). Criteria-based content analysis – A qualitative review of the first 37 studies. Psychology Public Policy and Law.

[b0150] Whalley, M. G., Kroes, M., Rugg, M. D., & Brewin, C. R. (submitted for publication). An fMRI investigation of posttraumatic flashbacks. 10.1016/j.bandc.2012.10.002 PMC3549493 23207576

